# Fast Mapping of Short Sequences with Mismatches, Insertions and Deletions Using Index Structures

**DOI:** 10.1371/journal.pcbi.1000502

**Published:** 2009-09-11

**Authors:** Steve Hoffmann, Christian Otto, Stefan Kurtz, Cynthia M. Sharma, Philipp Khaitovich, Jörg Vogel, Peter F. Stadler, Jörg Hackermüller

**Affiliations:** 1 Bioinformatics Group, Department of Computer Science, University of Leipzig, Leipzig, Germany; 2 Interdisciplinary Center for Bioinformatics, University of Leipzig, Leipzig, Germany; 3 Center for Bioinformatics, University of Hamburg, Hamburg, Germany; 4 Max Planck Institute for Infection Biology, Berlin, Germany; 5 RNomics Group, Fraunhofer Institute for Cell Therapy and Immunology IZI, Leipzig, Germany; 6 Santa Fe Institute, Santa Fe, New Mexico, United States of America; 7 Department of Theoretical Chemistry, University of Vienna, Vienna, Austria; 8 Max-Planck-Institute for Mathematics in Sciences, Leipzig, Germany; 9 Comparative Biology Group, Partner-Institute for Computational Biology, Shanghai, China; Philadelphia, United States of America

## Abstract

With few exceptions, current methods for short read mapping make use of simple seed heuristics to speed up the search. Most of the underlying matching models neglect the necessity to allow not only mismatches, but also insertions and deletions. Current evaluations indicate, however, that very different error models apply to the novel high-throughput sequencing methods. While the most frequent error-type in Illumina reads are mismatches, reads produced by 454's GS FLX predominantly contain insertions and deletions (indels). Even though 454 sequencers are able to produce longer reads, the method is frequently applied to small RNA (miRNA and siRNA) sequencing. Fast and accurate matching in particular of short reads with diverse errors is therefore a pressing practical problem. We introduce a matching model for short reads that can, besides mismatches, also cope with indels. It addresses different error models. For example, it can handle the problem of leading and trailing contaminations caused by primers and poly-A tails in transcriptomics or the length-dependent increase of error rates. In these contexts, it thus simplifies the tedious and error-prone trimming step. For efficient searches, our method utilizes index structures in the form of enhanced suffix arrays. In a comparison with current methods for short read mapping, the presented approach shows significantly increased performance not only for 454 reads, but also for Illumina reads. Our approach is implemented in the software segemehl available at http://www.bioinf.uni-leipzig.de/Software/segemehl/.

## Author Summary

The successful mapping of high-throughput sequencing (HTS) reads to reference genomes largely depends on the accuracy of both the sequencing technologies and reference genomes. Current mapping algorithms focus on mapping with mismatches but largely neglect insertions and deletions—regardless of whether they are caused by sequencing errors or genomic variation. Furthermore, trailing contaminations by primers and declining read qualities can be cumbersome for programs that allow a maximum number of mismatches. We have developed and implemented a new approach for short read mapping that, in a first step, computes exact matches of the read and the reference genome. The exact matches are then modified by a limited number of mismatches, insertions and deletions. From the set of exact and inexact matches, we select those with minimum score-based E-values. This gives a set of regions in the reference genome which is aligned to the read using Myers bitvector algorithm [1]. Our method utilizes enhanced suffix arrays [2] to quickly find the exact and inexact matches. It maps more reads and achieves higher recall rates than previous methods. This consistently holds for reads produced by 454 as well as Illumina sequencing technologies.

## Introduction

Since the 454 pyrosequencing technology [3] has been introduced to the market, the need for algorithms that efficiently map huge amounts of reads to reference genomes has rapidly increased. Later, high throughput sequencing (HTS) methods such as Illumina [4] and SOLiD (Applied Biosystems) have intensified the demand. The development of read mapping methods decisively depends on specifications and error models of the respective technologies. Unfortunately, little is known about specific error models, and models are likely to change as manufactures are constantly modifying chemistry and machinery. Increasing the read length is a key aim of all vendors — tolerating a trade-off with read accuracy. In a recent investigation on error models of 454 and Illumina technologies, it has been shown that 454 reads are more likely to include insertions and deletions while Illumina reads typically contain mismatches [5],[6]. Currently available read mapping programs are specifically designed to allow for mismatches when aligning the reads to the reference genome. Most of the programs, e.g. MAQ [7], SOAP [8], SHRiMP [9] or ELAND (proprietary), use seeding techniques that gain their speed from pre-computed hash look-up tables. Some of these programs, in particular SOAP and MAQ, are specifically designed to map short Illumina or SOLiD reads. Longer sequences cannot be mapped by these tools. The matching models of MAQ, ZOOM [10], SOAP, SHRiMP, Bowtie [11], and ELAND focus on mismatches and largely neglect insertions and deletions. Indels are only considered during subsequent alignment steps but not while searching for seeds. With indels accounting for more than two thirds of all 454 sequencing errors, this is a major shortcoming for these kinds of reads [5]. Only PatMaN [12] and BWA [13] are able to handle a limited number of indels.

Mapping is aggravated by the manufacturers' overestimation of their read accuracies. While an overall error rate of 0.5% has been observed for 454, the error rate increases drastically for reads shorter than 80 bp and longer than 100 bp [5], leading to considerably larger error frequencies in real-life datasets. This implies that, sequencing projects aiming to find short transcripts such as miRNAs lose a substantial fraction of their data, unless a matching strategy is used that takes indels into account. In Illumina reads, error rates of up to 4% have been observed [6]. This differs significantly from Illumina's specification. Compared to 454, the frequency of indels is significantly lower. Moreover, differences between reads and reference genome might also occur due to genomic variations such as SNPs. We present a matching method that uses enhanced suffix arrays to compute exact and inexact seeds. Sufficiently good seeds subsequently trigger a full dynamic programming alignment. Our method is insensitive to errors and contaminations at the ends of a read including 3′ and 5′ primers and tags. The results section describes the basic ideas and an evaluation of our segemehl software implementing our method. The technical details of the matching model are described in the Methods section at the end of this contribution.

## Results

### Outline of the Algorithmic Approach

A read aligner should deliver the original position of the read in the reference genome. Such a position will be called the *true position* in the following. Optimally scoring local alignments of the read and the reference genome can be used to obtain a possible true position, but because an alignment of the read with the reference genome at the true position does not always have an optimal score according to the chosen scoring scheme, this method does not always work. Nevertheless, there are no better approaches available unless further information about the read is at hand.

**Figure 1:**
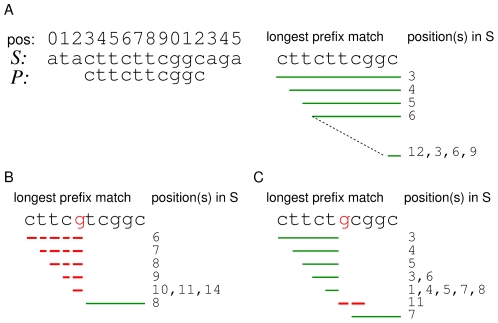
Longest prefix matches may fail to deliver the position of the optimally scoring local alignment. Assume a simple scoring scheme that assigns a score of +1 to a single character match and a score of 0 to a single character mismatch, a single insertions or deletion. Using longest prefix matches bears the risk of ignoring differences in the best, i.e. optimally scoring, local alignment. Its retrieval fails if a longer match can be obtained at another position of the reference sequence by matching a character, that is inserted, deleted, or mismatched in the best local alignment. Depending on the length of the reference genome and its nucleotide composition the probability is determined by the length of the substring that can be matched to the position of the best local alignment before the first difference occurs. (A) The optimally scoring alignment of the read *P*: = cttcttcggc begins at position 3 of the reference genome *S*: = atacttcttcggcaga. Let *P_i_* denote the *i^th^* suffix of the read *P*. For each *P_i_*, the starting positions of the longest match in *S* comprise the position of *P_i_* in the best local alignment (solid green lines). That is, the longest match of *P*_0_ begins at position 3, the longest match of *P*_1_ begins at position 4, the longest match of *P*_2_ begins at position 5 and so forth. (B) For the read *P*: = cttcgtcggc, the retrieval of the best local alignment fails for all *P_i_*, *i*<5 (dashed red line) due to the inclusion of a character that results in a mismatch in the optimally scoring local alignment. (C) The read *P*: = cttctgcggc contains, with respect to the best local alignment, a mismatch at position 5 of the read. Here the position 5 of the read is not included in the longest prefix match and nearly all substrings align correctly to the reference genome.

**Figure 2:**
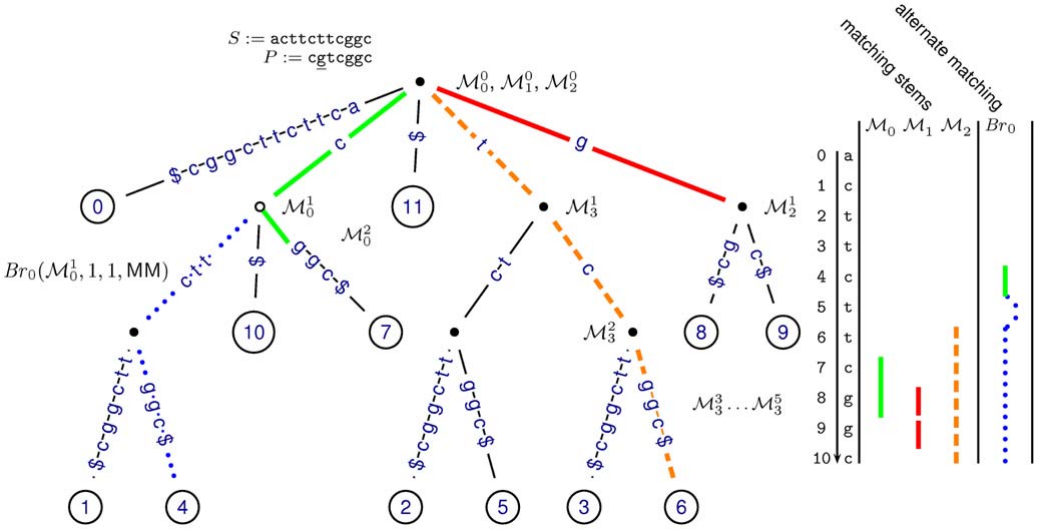
Matching stems and matching branches. We give an explanation based on a suffix trie which is equivalent to the suffix interval tree shown in Fig. 5 (see Methods). The suffix trie for *S*$ with *S*: = acttcttcggc (left) holds twelve leaves. Each numbered leaf corresponds to exactly one suffix in *S*. Nodes with only one child are not explicitly shown. Note, that internal nodes implicitly represent all leafs in their respective subtree. Thus, internal nodes can be regarded as sets of suffixes. The right panel holds the longest matches for different matching paths in the trie. Matching the first three suffixes of the read *P*: = cgtcggc results in three different paths in the suffix trie. Each path is equivalent to a sequence of suffix intervals, a matching stem, in the enhanced suffix array. Let 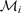 denote the matching stem for *P_i_* = *i^th^* suffix of *P*. The *q^th^* interval in 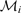, denoted by 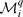, implicitly represents the set of suffixes in *S* matching *P*[*i*‥*i*+*q*−1]. The path for the first suffix *P*_0_ is of length two (green solid line). Hence, the equivalent matching stem 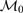 is a sequence of three intervals: 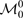, 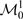 and 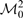. Since 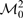 only represents the suffix *S*_7_, the longest prefix match of *P*_0_ is of length 2 occurring at position 7 of the reference sequence (right panel). The matching stem 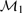 for *P*_1_ (red solid line) ends with 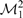. Therefore, matches of length one occur at positions 8 and 9 in *S*. The longest prefix match for *P*_3_ occurs at position 6 of *S* (dashed orange line). Note, that the intervals 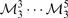 of 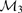 equivalently represent *S*_6_. An alternative path leads to a match with position 4. The branch 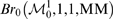 denotes the alternative that accepts the mismatch of *g* and *t* at position 1 of *P*_0_.

We present a new read mapping approach that aims at finding optimally scoring local alignments of a read and the reference genome. It is based on computing inexact seeds of variable length and allows to handle insertions, deletions (indels; gaps), and mismatches. Throughout the document the notion of differences refers to mismatches, insertions and deletions in some local alignment of the read and the reference genome, irrespective of whether they arise from technical artifacts or sequence variation. A single difference is either a single mismatch, a single character insertion or a single character deletion. Although not limited to a specific scoring scheme, we have implemented our seed search model in the program segemehl assigning a score of 1 to each match and a score of −1 to each mismatch, insertion or deletion. Our matching strategy derives from a simple and commonly used idea. Assume an optimally scoring local alignment of a read with the reference genome with exactly two differences. If the positions of the differences in the alignment are sufficiently far apart, we can efficiently locate exact seeds which in turn may deliver the position of the optimal local alignment in the reference genome. Likewise, if the distance between the two differences is small, two continuous exact matches at the ends of the read possibly allow to map the read to this position. To exploit this observation, the presented method employs a heuristic based on searches starting at all positions of the read. That is, for each suffix of the read the longest prefix match, i.e. the longest exact match beginning at the first position of the suffix with all substrings of the reference genome is computed. If the longest prefix match is long enough that it only occurs in a few positions of the reference genome, it may be feasible to check all these positions to verify if the longest prefix match is part of a sufficiently good alignment. While this approach works already well for many cases, we need to increase the sensitivity for cases where the computation of the longest prefix match fails to deliver a match at the position of the optimally scoring local alignment. This is the case when a longer prefix match can be obtained at another position of the reference genome by exactly matching characters that would result in a mismatch, insertion or deletion in the optimal local alignment (cf. Fig. 1). Therefore, during the computation of each longest prefix match we check a limited number of differences by enumerating at certain positions all possible mismatches and indels (cf. Fig. 2).

To efficiently compute the longest prefix matches, we exploit their properties for two consecutive suffixes of a read, i.e. for two suffixes starting at position *i* and *i*+1. If the suffix starting at position *i* has a longest prefix match of length *ℓ*, *then the suffix starting at position i+1 has a longest prefix match of length at least* *ℓ*−1. For example, assume a read ACTGACTG. If the second suffix has a longest prefix match of length 4, i.e. CTGA, with the reference genome, we immediately see that the third suffix has a longest prefix match not shorter than 3—because we already know that the substring TGA exists in the reference genome. Using an enhanced suffix array of the reference sequence, we can easily exploit this fact and determine the longest prefix match of the next suffix without rematching the first *ℓ* *−1 characters. Likewise, the enumeration of mismatches and indels is also restricted to the remaining characters of the suffix in our model.*

For each suffix of a read, we thus obtain a set of exact matches and alternative inexact matches and their respective positions in the reference sequence. These exact and inexact matches act as seeds. If a seed occurs more than *t* times in the reference genome, then it is omitted, where *t* is a user specified parameter (segemehl option –maxocc). The heuristics rigorously selects the exact or inexact seed with the smallest E-value, computed according to the Blast-statistics [14]. If this E-value is smaller than some user defined threshold (segemehl option -E), the bitvector algorithm of [1] is applied to a region around the genomic position of the seed to obtain an alignment of the read and the reference sequence. While the score based search for local alignment seeds controls the sensitivity of our matching model, the bitvector alignment controls its specificity: if the alignment has more matching characters than some user specified percentage *a* of the read (segemehl option -A) the corresponding genomic position is reported (see Methods).

The computation of the longest prefix match is implemented by a top-down traversal of a conceptual suffix interval tree, guided by the characters of the read. The suffix interval tree is equivalent to a suffix trie (see Methods). The traversal delivers a matching stem. Note that for the DNA alphabet there are at most four edges outgoing from each node of the suffix interval tree. To introduce mismatches, the traversal is simply continued with alternative edges, i.e. edges diverging from the matching stem. To introduce insertions, the traversal is not regularly continued, but characters of the read are skipped. Deletions are simulated by skipping nodes of the suffix interval tree and continuing the search at their child nodes (see Methods). We refer to these alternative paths that branch off from the matching stem as branches. The maximum number of branches to be considered is controlled by the seed differences threshold *k* (segemehl option -D). Note, that while matching character by character along a suffix of a read, the number of branches is expected to decrease quickly.

### Performance Tests

segemehl constructs indices either for each chromosome of a genome and the matching is performed chromosome-wise or, depending on the available RAM, chromosomes are combined to larger sequences. Compared to other methods, the index structure used by segemehl is significantly larger. For example, the enhanced suffix array of human chromosome 1 occupies approximately 3 GB of space. As it is stored on disk, the index only needs to be computed once. The construction of the index requires linear time. For example, on a single CPU, the construction of the complete enhanced suffix array for human chromosome 1 takes approximately 15 minutes. For our comparison, we ran segemehl with maximum occurrence parameter *t* = 500. The maximum E-value for seeds was set to 0.5 and minimum identity threshold to *a* = 85% which corresponds to a maximum of ⌈0.15·*m*⌉ differences in an alignment of the read of length *m*.

We compared segemehl to Bowtie v0.9.7 with option –all, BWA v0.2.0, MAQ v0.7.1, PatMaN v1.2.1 and SOAP v1.11 with option –r 2. MAQ and SOAP are based on ungapped alignments which are computed by hash lookups [7],[8],[13]. Due to length restrictions, MAQ is limited to Illumina (and SOLiD) reads. It additionally takes quality scores into account. The quality values needed by MAQ were, for all nucleotides, uniformly set to a value corresponding to the error rate. Bowtie [11] and BWA [13] index the reference genome with the Burrows-Wheeler transform. BWA allows a limited number of indels. PatMaN [12] matches the reads by traversing a non-deterministic suffix automaton constructed from the reference genome. Except for PatMaN, all programs only report matches with the smallest edit distance. BWA and Bowtie each need about 10 minutes to build their index. The fastq files needed by MAQ are built in approximately 2 minutes. PatMaN and SOAP require no indexing steps. The options for the other programs were chosen so as to achieve results similar to segemehl. For our comparison, we performed tests on simulated as well as real-life read data sets. For the simulation we generated read sets representing different error rates, types and distributions. We used three distinct error sets, one containing only mismatches, one containing only indels and a last one representing reads with mismatches and indels at a ratio of 1∶1. Additionally, different error distributions were used to model error scenarios such as terminal contamination (e.g. linker, poly-A tails) or decreasing read quality. We chose uniform, 5′, 3′ and terminal error distributions.

Each simulated dataset contained 500 000 simulated reads, each of length 35 bp, sampled from a 50 MB large region of the human genome (chromosome 21). We introduced errors to each simulated read according to previously defined rates, error types and distributions. For the 50 MB region we constructed the indexes required for segemehl and Bowtie. For MAQ we constructed the index for the read set under consideration. Index construction took approximately one minute for Bowtie and BWA. The construction for the enhanced suffix array for segemehl took 3.5 minutes. The binary fastq files for MAQ were created in about 20 seconds.

**Figure 3:**
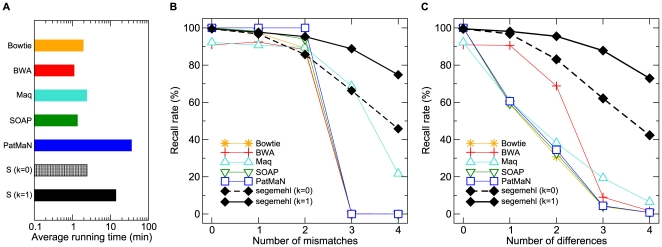
Comparison of recall rates and running time for several short read aligners. Average running time for the different programs (A) in matching runs with 500 000 reads in two different data sets (logarithmic scale; S refers to segemehl). The differences are uniformly distributed and consist of only mismatches (B) or mismatches, insertions and deletions (C). The recall rate describes the fraction of reads which was mapped to the correct position. All programs were used with default parameters. Bowtie was called with option –all and SOAP with option –r 2.

We ran segemehl with seed differences threshold *k* = 0 and *k* = 1. For *k* = 0, only exact seeds are computed and for *k* = 1 seeds with at most one difference are computed. All programs were executed single-threaded on the same machine. The results for a uniform error distribution for mismatches only as well as for mismatches and indels are shown in Fig. 3. We measured the performance in terms of running time (Fig. 3 (A)) and recall rates, i.e. the percentage of reads mapped to the correct position. segemehl has recall rates of more than 95% (*k* = 1) and 80% (*k* = 0) in each setup with not more than two errors in the reads. With four uniformly distributed errors in the reads, the recall rate drops below 80% (*k* = 1) and 50% (*k* = 0), respectively. Hence, for *k* = 1 segemehl outperforms all other methods in terms of recall rates. For reads containing only mismatches and *k* = 0, segemehl is comparable to other methods (Fig. 3 (B)) while it has a significantly better recall rate as soon as insertions and deletions are involved (Fig. 3 (C)). As expected, the recall rate of most short read aligners drops if insertions and deletions are introduced into the reads. The running time of segemehl for *k* = 0 is comparable to other short read aligners. For *k* = 1, the running time increases by a factor of 10.

**Figure 4:**
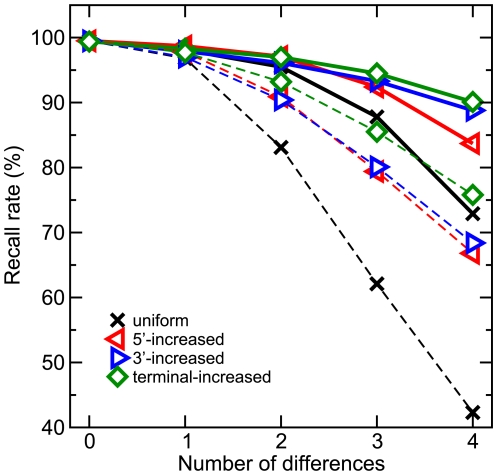
segemehl recall rates for varying difference values and distributions. Recall rates are depicted for *k* = 0 (dashed) and *k* = 1 (solid). For terminal–, 3′– and 5′– increased difference distributions, segemehl achieves a recall rate above 80% for reads with 4 errors.

In contrast to Bowtie, BWA, MAQ, and SOAP, segemehl reports, by default, multiple matches for a read within the reference genome if the corresponding alignments have an E-value smaller than some user defined threshold. This behavior leads to an increase in the running time and a decrease in specificity. Compared to PatMaN, which is also able to report multiple matches, segemehl can cope with more than two differences and still is on average faster by a factor of 1.7 (*k* = 1) and 14 (*k* = 0). As expected, the worst segemehl results are seen for high error rates with a uniform error distribution (Fig. 4). Terminal, 3′ and 5′ error distributions yield better results, suggesting that segemehl implements a robust method that is insensitive to leading and trailing contaminations. Next, we compared segemehl, Bowtie and MAQ on two real-life data sets. We used Bowtie with option –all and MAQ with option –C 513 as suggested in the manuals to achieve maximum sensitivity. segemehl's sensitivity was controlled by option –M 500 to omit all seeds occurring more than 500 times in the reference sequence.

The data set ERR000475 of 20 million Illumina reads (length 45) for *H. sapiens* was downloaded from the NCBIs Short Read Archive (http://www.ncbi.nlm.nih.gov/Traces/sra/). The second data set comprised about 40 000 short 454 reads from the *arabidopsis mpss plus database* (http://mpss.udel.edu/at/). The average length of the 454 reads was 23 bp.

We partitioned the 454-set into subsets of equal size, to satisfy input requirements for MAQ. An average quality value was assigned to each base.

Mapping multiple reads to a reference genome is a task which can easily be parallelized. Like all other methods, segemehl offers a parallelization option to run the program on multiple cores. segemehl runs for the ERR000475 dataset were carried out in eight parallel threads on a single machine with two Quadcore CPUs and 16GB of RAM. Seven enhanced suffix arrays were constructed representing the whole human genome. segemehl mapped 92% of the reads to the reference sequence while MAQ mapped 85% without and 89% with quality values. The corresponding values for Bowtie are 81% and 89%. The largest difference between the three tools is for the total number of exact matches. Although MAQ was, according to the manual, running in maximum sensitivity mode, segemehl computes 20 times more matches than MAQ (Tab. 1 (a)). Bowtie reports 2.5 billion matches which is much more than the two other tools. As expected, for the 454-set, the difference among the compared programs is even larger. While Bowtie is able to map 71% of all reads, segemehl achieves 95%. MAQ, a program explicitly designed for Illumina reads, matches 79% of the reads. Interestingly, compared to Bowtie, MAQ reports more matches with two mismatches. segemehl mainly achieves this result by mapping more reads with one or two errors. In fact, by allowing insertions and deletions segemehl doubles the number of reads matched at the unit edit distance of 1 (Tab. 1 (b)).

**Table 1:** Comparison of the performance of Bowtie, MAQ, and segemehl on two real-life datasets.

			total	mismatches+insertions+deletions			
				0	1	2	≥3
(a) Human genomic data set ERR000475 (Illumina)							
	Bowtie (-all)	all matches	2 692 341 844	631 194 732	925 094 123	1 136 952 989	-
		reads found	16 011 867 (81%)	12 006 627	2 824 359	1 180 881	-
	Bowtie (-all) with qualities	all matches	9 264 604 839	631 194 732	914 965 615	1 098 260 521	6 620 183 971
		reads found	17 693 135 (89%)	12 006 627	2 806 842	1 162 905	1 716 761
	MAQ	all matches	67 108 174	22 545 585	15 999 878	14 913 062	13 649 649
		reads found	16 762 361 (85%)	12 006 627	2 829 601	1 199 110	727 023
	MAQ with qualities	all matches	96 980 574	15 084 354	9 867 729	10 987 486	61 041 005
		reads found	17 725 314 (89%)	11 277 928	2 928 839	1 364 477	2 154 070
	segemehl	all matches	701 943 169	464 294 770	112 471 308	42 794 605	57 262 900
		reads found	18 191 858 (92%)	12 002 123	2 872 615	1 221 313	2 095 807
(b) *A. thaliana* short RNA data set (454)							
	Bowtie (-all)	all matches	156 621	85 254	42 443	28 924	-
		reads found	26 969 (71%)	18 739	5 390	2 840	-
	MAQ	all matches	74 994	26 890	15 078	14 482	18 544
		reads found	29 987 (79%)	18 738	5 389	3 093	2 767
	segemehl	all matches	262 262	72 328	83 070	51 048	55 816
		reads found	35 942 (95%)	18 737	10 525	3 744	2 936

(a) The genomic paired DNA library with 19 812 604 Illumina reads was matched using MAQ (chromosome by chromosome), Bowtie (single index), and segemehl (seven enhanced suffix arrays each representing a disjoint subset of the human chromosomes). Bowtie was used with and without quality values. To simulate a MAQ run without quality information, an average quality value was assigned to all bases of the Illumina data set. The total number of matches differs significantly: Bowtie outnumbers all other programs. segemehl still reports ten times more matches than MAQ without quality values. The number of exact matches shows a 20-fold increase. Although MAQ improves when quality values are used, the total number of matches remains small in contrast to the other programs. Differences in the number of exactly matching reads reflect the distinct handling of repetitive and uninformative reads. In segemehl, all reads matching more often than *t* = 500 times are dropped. (b) 38171 reads of a small short RNA library sequenced with 454 were matched to the *A. thaliana* genome. Compared to Bowtie and MAQ, segemehl mapped significantly more reads. Allowing for one error, segemehl matches twice as many reads as Bowtie, due to the fact that segemehl, unlike Bowtie, allows for indels. Note that segemehl discarded a few perfect matches since the corresponding seeds occur more than *t* = 500 times in the reference sequence.

## Discussion

We have presented a novel read mapping approach that is able to efficiently handle 3′ and 5′ contaminations as well as mismatches, insertions and deletions in short and medium length reads. It is based on a matching model with inexact seeds containing mismatches, insertions and deletions. The sensitivity and specificity of our method is controlled by a maximum seed differences threshold, a maximum occurence threshold, an E-value threshold and an identity threshold. Compared to previous methods, our approach yields improved recall rates especially for reads containing insertions and deletions. Since indels have been reported to be the predominant error type in 454 reads, allowing for indels is most important to achieve a correct mapping. While PatMaN, by default, fully enumerates all matches with up to two differences, segemehl's heuristic reports only best-scoring matches. The price for the gain in sensitivity is an increase in running time: with *k* = 1 our method is approximately ten times slower than Bowtie, the fastest program in our comparison. As we used enhanced suffix arrays, matching against a large mammalian genome has to be done chromosome by chromosome when off-the-shelf hardware is used. However, the gain in sensitivity for reads with mismatches and the failure of other methods when dealing with indels may be, depending on the users demands, a reasonable trade off for these shortcomings. Our method is not limited to a specific technology or read length. Although quality values are not considered yet, the matching strategy can easily be adapted to evaluate low quality bases specifically. In principle, we show that for *k* = 0, i.e. exact seeds, our method is sufficiently sensitive to map reads with up to two differences. This is an interesting result since most of the current methods do not tolerate insertions and deletions. In summary, segemehl with *k* = 0 is among the fastest mapping algorithms. For *k* = 1, segemehl is able to achieve good recall rates beyond the two error barrier. This is especially interesting since manufacturers try to increase their read lengths at the cost of higher error rates. The increased sensitivity of the presented matching model, along with its ability to handle leading and terminal contaminations is a trade off for the large memory requirements of the enhanced suffix arrays. In the future, compressed index structures like the FM-index [15] may be a suitable framework to implement our matching model with smaller memory requirements.

## Methods

Our strategy, based on enhanced suffix arrays, aims to find a best local alignment of short reads and reference sequences with respect to a simple scoring system. It does so by determining, for each suffix of the read, the longest prefix occurring as a substring in the reference sequence. This gives a matching backbone, from which a limited number of branches are derived by mismatches, insertions and deletions (Fig. 2). The concept of a matching backbone is equivalent to the concept of matching statistics introduced in [16]. We introduce the concept of matching backbone and branches via a conceptual tree of suffix intervals. Our heuristic approach delivers a small number of inexact seeds of variable length that are subsequently checked by the bitvector algorithm of Myers [1] to verify the existence of alignments with a limited number of differences. First, a short introduction to the basic notions for sequence processing and enhanced suffix arrays will be given, before the concept of suffix intervals is defined. Subsequently, we introduce our new matching strategy.

### Basic Notions for Sequence Processing

We consider sequences over the DNA alphabet Σ_DNA_ = {A, C, G, T, N}, where N denotes an undetermined base. In our approach the alignment of N with any character, including N itself, results in a mismatch.

#### Enhanced suffix arrays

First we introduce basic notions for the suffix array and enhanced suffix array. We then formally introduce the concept of a suffix interval.

Suppose that *S* is a sequence of length *n*. We index *S* from position 0. That is, *S*[*i*] denotes the character at position *i* in *S*, for 0≤*i*≤*n*−1. For *i*≤*j*, *S*[*i*‥*j*] denotes the substring of *S* starting with the character at position *i* and ending with the character at position *j*. For *i*>*j*, *S*[*i*‥*j*] denotes the empty string. *occ_S_*(*w*) denotes the set of occurrences of some string 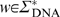 in *S*, i.e. the set of positions *i*, 0≤*i*≤|*S*|−|*w*| satisfying *w* = *S*[*i*‥*i*+|*w*|−1]. A substring of *S* beginning at the first position of *S* is a prefix of *S* and a substring ending at the last position of *S* is a suffix of *S*. To prevent that suffixes have a second occurrence in *S*, we add a sentinel character $ (not occurring in *S*) to the end of *S*. For each *i*, 0≤*i*≤*n*, *S_i_* = *S*[*i*‥*n*−1]$ denotes the *i*-th non-empty suffix of *S*$, i.e. the suffix beginning at position *i* in *S*$. We identify a suffix of *S*$ by its start position. That is, by suffix *i* we mean *S_i_*.

The concept of suffix arrays is based on lexicographically sorting the suffixes of *S*$. Suppose that the characters are ordered such that A<C<G<T<N<$. This character order induces an order on all non-empty suffixes of *S*$, which is captured in the suffix array. Formally, the suffix array suf of *S* is an array of integers in the range 0 to *n*, specifying the lexicographic order of the *n*+1 non-empty suffixes of *S*$. In other words, *S*_suf[0]_, *S*_suf[1]_, …, *S*_suf[*n*]_ is the sequence of suffixes of *S* in ascending lexicographic order.

The lcp-table lcp is an array of integers in the range 0 to *n*−1. For each *h*, 1≤*h*≤*n*, lcp[*h*] is the length of the longest common prefix of *S*_suf[*h*−1]_ and *S*_suf[*h*]_. Since the suffix *S_n_* = $ is the last suffix in the lexicographic order of all non-empty suffixes, *S*_suf[*n*]_ = $. Hence we always have lcp[*n*] = 0. The enhanced suffix array is the combination of the suffix array, the lcp-table and two other tables from [2] not defined here, namely the child-table and the suffix link table.

We now formally introduce the notion of *suffix intervals* that is at the heart of our matching strategy in enhanced suffix arrays.

An interval [*l*‥*r*, *h*] is a *suffix interval* if the following holds:

1. 0≤*l*≤*r*≤*n*
2. 0≤*h*≤*n*+1
3. lcp[*i*]≥*h* for all *i*, *l*+1≤*i*≤*r*
4. *l* = 0 or lcp[*l*]<*h*
5. *r* = *n* or lcp[*r*+1]<*h*

A suffix interval [*l*‥*r*, *h*] refers to table suf, denoting the set *ϕ*([*l*‥*r*, *h*]) = {suf[*j*] |*l*≤*j*≤*r*} of suffixes of *S*$. *l* and *r* are the interval boundaries of [*l*‥*r*, *h*]. We say that suffix *S_i_* is in the suffix interval [*l*‥*r*, *h*] if *i*∈*ϕ*([*l*‥*r*, *h*]). *r*−*l*+1 is the *width* of [*l*‥*r*, *h*].

All suffixes of *S*$ in a suffix interval [*l*‥*r*, *h*] have a common prefix, say *w*, of length *h*. Vice versa, all suffixes of *S*$ having prefix *w* are in [*l*‥*r*, *h*]. Due to this correspondence, we say that [*l*‥*r*, *h*] *is the suffix interval for w*. Note that *ϕ*([*l*‥*r*, *h*]) = *occ_S_*(*w*) whenever [*l*‥*r*, *h*] is the suffix interval for *w*.

**Figure 5:**
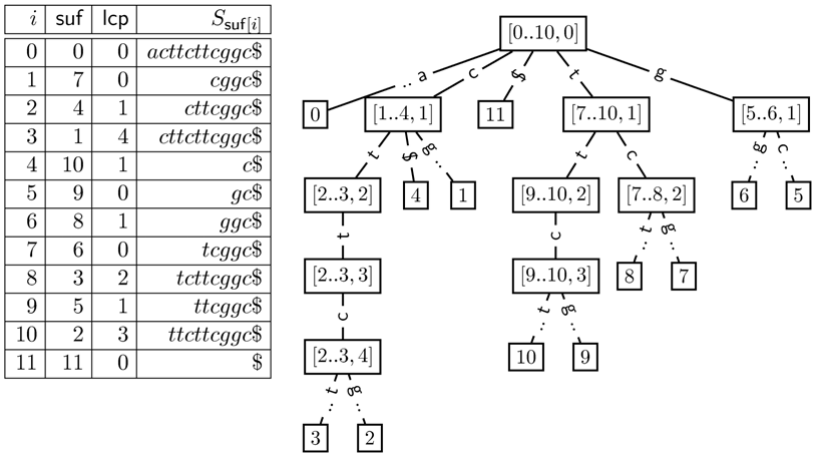
The enhanced suffix array yields a tree structure of nested suffix intervals. The enhanced suffix array for the sequence *S*: = attcttcggc (left) and its suffix interval tree (right), equivalent to the suffix trie in Fig. 2, is shown. The array suf represents the lexicographical order of the suffixes in *S*$. In other words, *S*_suf[0]_, *S*_suf[1]_, …, *S*_suf[*n*]_ is the sequence of suffixes of *S*$ in ascending lexicographic order. The lcp-table lcp is an array of integers such that for each *h*, 1≤*h*≤*n*, lcp[*h*] is the length of the longest common prefix of *S*_suf[*h*−1]_ and *S*_suf[*h*]_. A suffix interval [*l*‥*r*, *h*] denotes an interval in the suffix array with lcp[*i*]≥*h* for all *i*, *l*+1≤*i*≤*r*, i.e. all suffixes in the interval [*l*+1‥*r*] have a longest common prefix of length at least *h*. Additionally, requiring *l* = 0 or lcp[*l*]<*h* makes the suffix interval left maximal and requiring *r* = *n* or lcp[*r*+1]<*h* makes it right maximal. The suffix interval [0‥10, 0] spans the whole suffix array and is equivalent to the root of a suffix interval tree. This interval contains five subintervals, one for each character in *S*$, with *h* = 1. Equivalently, the root node of the suffix interval tree has five children. Note, that two children, labeled by 0 and 11, are singletons. The child nodes of singletons are not explicitly shown here.

The notion of suffix intervals slightly generalizes the notion of lcp-intervals, as introduced in [2]. A suffix interval [*l*‥*r*, *h*] of width at least 2 is an *lcp-interval* if, besides condition 1.–5. above, we additionally have lcp[*i*] = *h* for at least one *i*, *l*+1≤*i*≤*r*. This condition requires that at least one pair of consecutive suffixes in the suffix interval has a longest common prefix of length exactly *h* (Fig. 5). In other words, a suffix interval [*l*‥*r*, *h*] of width 2 which is not an lcp-interval does not have a maximum lcp-value *h*, implying that [*l*‥*r*, *h*+1] is also a suffix interval.

While suffix intervals correspond one-to-one to the nodes of a suffix trie for *S*$ (cf. [17]), lcp-intervals correspond to the branching nodes of a suffix tree for *S*$ (cf. [2]). Interpreting the additional condition for lcp-intervals for trees means that in suffix trees nodes with a single child are omitted, while they are allowed in suffix tries.

### Matching Concept

Consider the suffix interval [*l*‥*r*, *h*] for *w*. A child of [*l*‥*r*, *h*] is a suffix interval [*l*′‥*r*′, *h*+1] satisfying *l*≤*l*′≤*r*′≤*r*. We call [*l*′‥*r*′, *h*+1] the a-child of [*l*‥*r*, *h*] if there is a character 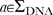 such that [*l*′‥*r*′, *h*+1] is the suffix interval for 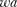. Note that for all *q*, *l*′≤*q*≤*r*′, we have 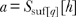 = *S*_suf[*q*]_[*h*]. Hence we can easily determine 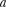 from [*l*′‥*r*′, *h*+1] or split [*l*‥*r*, *h*] into its children. A method computing the *a*-child of a suffix interval in constant time is described in [2].

Let 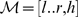 be a suffix interval. For the empty sequence *ε* we define 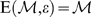. For any character 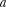 and any sequence *u* we recursively define
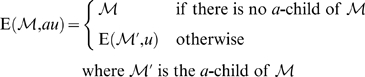



That is, 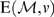 delivers the interval 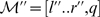, obtained by greedily matching the characters in *v* beginning at the suffix interval 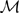 and *q* is the length of the matching prefix of *v*.

Let *P* denote a sequence of length *m* neither containing a wildcard symbol N nor the sentinel $. For any *i*, 0≤*i*≤*m*, *P_i_* = *P*[*i*‥*m*−1] denotes the suffix of *P* beginning at position *i*. Let *ℓ*_i_ be the length of the longest prefix of *P_i_* occurring as a substring of *S*. Then P[i‥i+*ℓ* *_i_*−1] occurs in *S* and either *i*+*ℓ*_i_ = m *or* P[i‥i+*ℓ* *_i_*] does not occur in *S*. Moreover, there is a sequence of suffix intervals 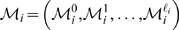, such that for all *q*, 0≤*q*≤*ℓ* *_i_*, 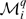 is the suffix interval for *P*[*i*‥*i*+*q*−1]. This implies that 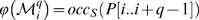. We call 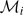 a matching stem. Obviously, for any *i*, 0≤*i*≤*m*, 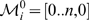. For any *i*, 0≤*i*≤*m* and any *q*, 1≤*q*≤*ℓ*_i_, 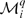 is the 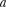-child of 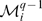 where 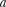 = *S*[*t*+*q*−1] for any 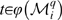. (Note that all suffixes in 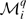 have the common prefix *P*[*i*‥*i*+*q*−1] and 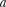 is the last character of this prefix.) The *ℓ*_i_-values are determined in the same way as the length-values of the matching statistics, introduced in [16]. Using the suffix link table, the *ℓ*_i_-values can be computed in *O*(*m*) time altogether (cf. [2]).

We now consider the relation of matching stems of two neighboring suffixes *P_i_*_−1_ and *P_i_* for some *i*>0. First note that *ℓ*_i__*−1*_≤*ℓ* *_i_*+1. Moreover, for each *q*, 1≤*q*≤*ℓ*_i__*−1*_ we have 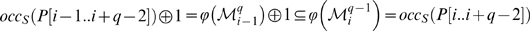
where 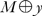 = (x+y | x∈M} denotes the elementwise addition for any set *M*. That is, any suffix in 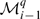 can be found in 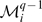 with offset one.

To allow differences in our matching heuristic, we introduce the concept of matching branches which branch off from sets of the matching stem. We describe the branching in terms of a transformation of some suffix interval 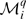.

Let *i*, 0≤*i*≤*m*−1 be arbitrary but fixed. Let *q* be such that *i*+*q*−1<*m*. Consider some suffix interval 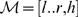 such that the unit edit distance of *S*[suf[*l*]‥suf[*l*]+*h*−1] and *P*[*i*‥*i*+*q*−1] is exactly *d*≤*k*. Then, for the edit operations *x*∈{MM, I, D}, we define the matching branch 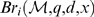 as follows:
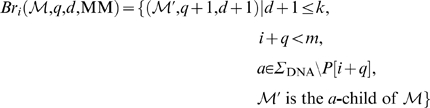


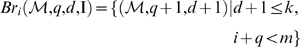


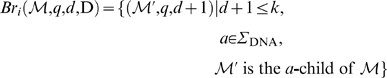



Any computation of a triple (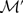, *q*′, *d*+1) according to these equations is called branching step. The MM-branching step implies a mismatch of *a*≠*P*[*i*+*q*] (in the reference sequence) with *P*[*i*+*q*] (in the read). The I-branching step implies an insertion of character *P*[*i*+*q*] in the read. The D-branching step implies a deletion of character *a*∈Σ_DNA_ in the read.

Note that in case some *a*-child of 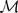 does not exist, there is no corresponding contribution to the matching branch. We combine the different types of matching branches by defining:
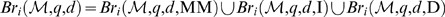



Obviously, any element in 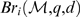 can itself be extended by branching from it. To define this, we introduce for all *j*≥1 the iterative matching branch 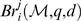 as follows:
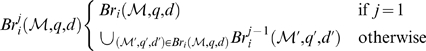



This gives us the matching branch closure 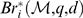, defined by
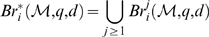



**Figure 6:**
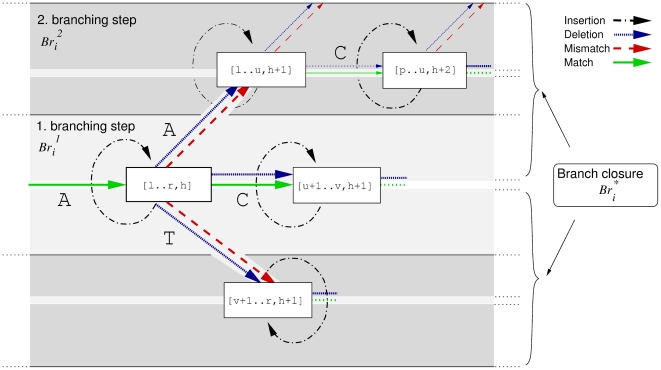
The branch closure. The suffix interval [*l*‥*r*, *h*], representing some string 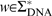 of length *h*, is split into its children [*l*‥*u*, *h*+1], [*u*+1‥*v*, *h*+1] and [*v*+1‥*r*, *h*+1] by matching an additional character *a*∈{A, C, T}. We proceed building 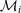 by matching the character C (solid bold green line). Beforehand, alternative suffix intervals are stored in 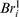, either representing mismatches (dashed red line), insertions (dashed dotted black line) or deletions (dotted blue line). 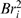 holds suffix link intervals that in turn branch off from 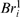. The branch closure 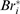 holds all such alternative intervals.

That is, 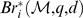 is the set of matching branches that can be derived by one or more branching steps from (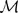, *q*, *d*) (Fig. 6). Of course, since each step increases the difference value *d*, the number of steps is limited by *k* – *d*. Each element 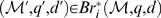 is extended by exactly matching *P*[*i*+*q*′‥*m*−1] against the enhanced suffix array beginning at the suffix interval 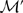. That is, we compute 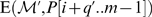.

While we have defined matching branches for any element in a matching stem, we only compute them for a few elements of the matching stem which make up the matching backbone: Let 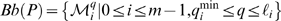, where 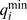 is defined by
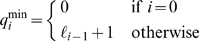



**Figure 7:**
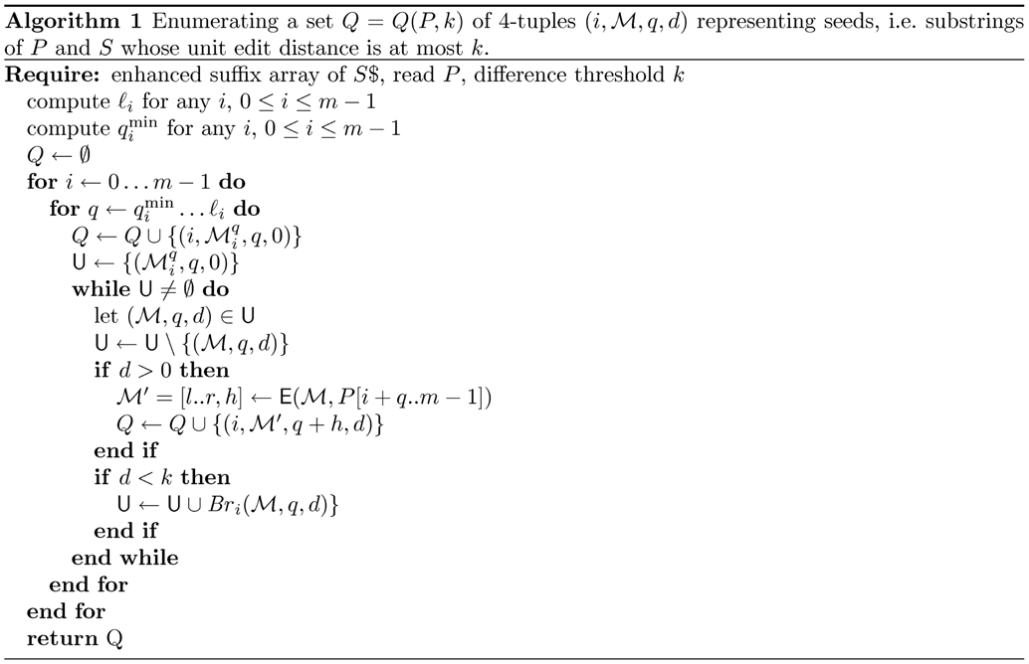
Algorithm. Enumeration of exact and inexact seeds.

Thus, for each suffix *i*, 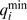 is the position in *P* from which to continue processing the next suffix. For any 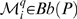, we compute 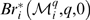. That is, we omit computing 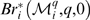 for 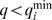. This is due to the fact that some of the suffixes in 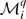 are already included (with offset one) in 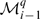, see equation (1). All in all, we arrive at a set *Q*(*P*, *k*) of 4-tuples (*i*, 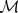, *q*, *d*) such that the unit edit distance of *P*[*i*‥*i*+*q*−1] and *w* is *d*≤*k* and 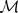 is the suffix interval for *w*. The Figure 7 gives pseudocode for computing *Q*(*P*, *k*) (which includes the matching backbone).

Turning to the analysis of the algorithm, first note that
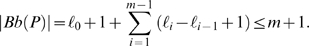



That is, the matching backbone contains at most *m*+1 elements and thus the statements in the inner loop of the algorithm (Fig. 7) are executed *O*(*m*) times altogether. Obviously 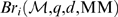 contains up to 5 elements, 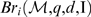 contains at most 1 element and 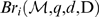 contains at most 6 elements. Since there can be *k* iterations when computing 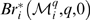, the size of this set is at most (12)*^k^*. Hence the total number of all matching branches is (*m*+1) · (12)*^k^*. Each matching branch is generated from a previously generated element in constant time. Hence the algorithm runs in time proportional to (*m*+1) · (12)*^k^*.

From the matching backbone and from the set of all matching branches we select an element achieving a maximum score according to a simple scoring scheme where a character match scores +1 and a mismatch, an insertion and a deletion scores −1. The maximum score element (*i*, [*l*‥*r*, *h*], *q*, *d*) defines a set of substrings of *S* which are aligned to *P*. More precisely, for any *j*, *l*≤*j*≤*r*, *P* is matched against the reference substring *S*[suf[*j*]−(*i*+*k*)‥suf[*j*]+(*m*−*i*+*k*)] using the bit vector algorithm of Myers [1]. For this, we allow a maximum number 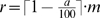 of differences, according to the the identity threshold 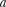. Myers algorithm runs in *O*(*m*/*ω* · *ℓ*) time where *ℓ* = 2*k*+*m*+1 is the length of the reference substring and *ω* is the word size of the machine. As *ω* = 64 in our implementation, for reads of size up to 64, we have *m*/*ω* = 1 and so the algorithm runs in *O*(*m*+*k*) time. Note that this running time is independent of *a*. In summary, by specifying *k* along with some E-value [14] we set the thresholds to search for local alignment seeds. Subsequently, we use Myers algorithm to discards all seeds that produce poor semi-global alignments, according to parameter *a*, typically loosely set to values around 80% (which corresponds to 
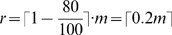
).
